# Prepubertal Unilateral Gynecomastia: Report of 2 Cases

**DOI:** 10.4274/jcrpe.1477

**Published:** 2014-12-05

**Authors:** Hüseyin Demirbilek, Gökhan Bacak, Rıza Taner Baran, Yahya Avcı, Ahmet Baran, Ayşenur Keleş, Mehmet Nuri Özbek, Yasemin Alanay, Khalid Hussain

**Affiliations:** 1 Diyarbakır Children State Hospital, Clinic of Pediatric Endocrinology, Diyarbakır, Turkey; 2 Great Ormond Street Hospital for Children, Clinic of Pediatric Endocrinology, London, United Kingdom; 3 University College, Institute of Child Health, London, United Kingdom; 4 Diyarbakır Training and Research Hospital, Clinic of Plastic and Reconstructive Surgery, Diyarbakır, Turkey; 5 Dicle University Faculty of Medicine, Department of Pathology, Diyarbakır, Turkey; 6 Acıbadem University Faculty of Medicine, Department of Pediatrics, Pediatric Genetics Unit, Istanbul, Turkey

**Keywords:** gynecomastia, pubertal, prepubertal, unilateral

## Abstract

Prepubertal unilateral gynecomastia is an extremely rare condition. At present, its etiology and management strategy are not well known. Two unrelated prepubertal boys of ages 8 and 9 who presented with complaints of unilateral enlargement of breast tissue are reported. Physical examination, biochemical, hormonal and oncologic work-up findings were normal. Both patients were treated with peripheral liposuction successfully. Histopathological and immunohistochemical examinations showed benign fibroglandular gynecomastia and intensive (3+) estrogen receptor expression in 100% of periductal epithelial cells. Although an extremely rare and generally benign condition, patients with prepubertal unilateral gynecomastia should have a full endocrine and oncologic work-up.

## INTRODUCTION

Gynecomastia is defined as a bilateral or unilateral enlargement of breast tissue in male subjects. It is highly prevalent in the neonatal period, puberty and in males over age 50 years (1,2). Gynecomastia in the neonatal period is usually due to intrauterine exposure to maternal estrogen (1). Increased androgen levels at puberty and concurrent increase in conversion of androgens to estrogens may cause pubertal gynecomastia (1). Pubertal gynecomastia is generally bilateral and in most cases physiological. However, cases with a breast enlargement ≥ grade 3 need a full endocrine and oncologic evaluation to exclude underlying pathologic disorders. In contrast to pubertal gynecomastia, prepubertal gynecomastia and especially unilateral prepubertal gynecomastia are extremely rare conditions with only a few case reports in the medical literature (2,3,4,5,6). Thus, there is lack of data on its etiology and management strategies. Herein, we present two unrelated boys with idiopathic prepubertal unilateral gynecomastia and their successful treatment with peripheral liposuction.

## CASE REPORTS

**Case 1**

**Figure 1:**
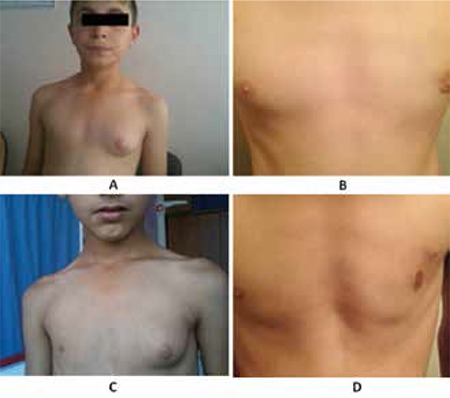
Unilateral gynecomastia before surgery (A&C) and at the 6-month follow-up visit post surgery (B&D)

A 9.8-year-old boy presented to our clinic with enlargement of his left breast tissue (Figure 1). He was otherwise healthy and his medical history was unremarkable. He had no history of exposure to exogenous systemic or topical estrogenic compounds. Breast development was first observed 2 years previously. At the time of presentation, his height was 143 cm [standard deviation (SD) score:0.99], weight was 41 kg (SD score:1.27) and body mass index (BMI) was 20.0 kg/m2 (SD score:1.07). He had grade 4 breast development on the left side (according to the adapted classification by The American Society of Plastic Surgeons) and normal breast tissue on the contralateral side. On assessment of his pubertal status, testes size was 3 mL by 3 mL and axillary/pubic hair development was prepubertal. There was no acceleration neither in somatic growth nor in bone age. Bone age was compatible with age 10 years. There was no other pathological or dysmorphic finding on physical examination. Hormonal evaluation (Table 1), liver and renal function tests, as well as scrotal, abdominal and contralateral pectoral region ultrasonography (US) were normal. Breast US showed a 54 x 15 mm fibroglandular tissue without any cystic or solid tumoral lesion. Etiologic evaluation did not show any pathological finding. A diagnosis of idiopathic unilateral prepubertal gynecomastia was considered.

**Case 2**

A 10.5-year-old boy presented with enlargement of the left breast tissue (Figure 1). His medical and family history was unremarkable. Breast development was first noticed 1.5 years before presentation. At the time of admission, his height was 136.4 cm (SD score:-0.50), weight was 27.2 kg (SD score:-1.29) and BMI was 14.6 kg/m2 (SD score:-1.43). There was no any other pathological finding on physical examination. His testes size was 2 mLx2 mL bilaterally. Axillary and pubic hair stages were prepubertal. He had grade 3-4 breast development on the left side, with normal contralateral breast tissue (according to the adapted classification by The American Society of Plastic Surgeons). There was no acceleration in somatic growth and bone age (bone age was 8 years). Hormonal evaluation, liver and renal function tests were normal (Table 1). Scrotal, abdominal and contralateral pectoral muscle US findings were normal as well. Breast US showed a fibroglandular tissue 40x10 mm in size, without any cystic or solid mass. This patient was also considered as a case of idiopathic unilateral prepubertal gynecomastia.

Both patients were also found to be in a depressed mood and with feelings of social isolation due to concerns about their feminine appearance. Therefore, a surgical excision was decided as the treatment of choice in both cases.

**Surgical Therapy**

Surgical resection was performed with peripheral liposuction technique with no perioperative complications. In addition, a remarkable improvement was observed in the psychological state of both patients at the post-surgical 6-month follow-up visit. There was also no evidence of recurrence (Figure 1).

**Histopathologic and Immunohistochemical (IHC) Findings of the Specimens Obtained at Surgery**

**Figure 2:**
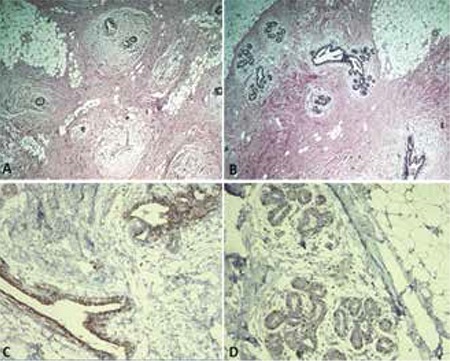
Histopathologic evaluation of resected breast tissue suggesting fibroglandular benign gynecomastia (A&B:H&E, x25), (C:CK5/6 and D:P63) (Immune peroxidase, x100)

The histopathological examinations of the formalin-fixed paraffin-embedded breast materials processed with standard methods and stained with hematoxylin and eosin (H&E) and immune peroxidase (CK5/6 and P63) revealed benign fibroglandular gynecomastia with no evidence of any pathological finding (Figure 2).

**Figure 3:**
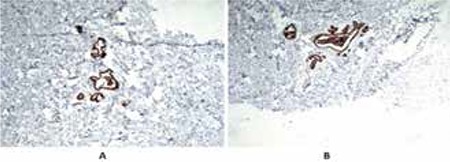
(A&B) Periductal intensive (3+), cytosolic and nuclear estrogen receptor (100%) staining of glandular tissue (x25)

For IHC examination, consecutive sections were processed. To show ER protein expression on IHC, paraffin-embedded sections, obtained from the 4 μm thick sections, were processed by standard methods, using primary monoclonal antibody (ER, Clone 6F11, Leica Microsystems Inc., IL, US) and Mayer’s hematoxylin protocol. IHC examination showed an intensive (3+) cytosolic and nuclear staining of estrogen receptor in 100% of periductal epithelial cells (Figure 3).

## DISCUSSION

Studies conducted on the etiology, therapy and follow-up of prepubertal gynecomastia are scarce and the underlying etiopathology is not known in up to 90% of cases. These cases are generally classified as idiopathic (7,8). In a study evaluating the largest pediatric cohort presenting with gynecomastia, Einav-Bachar et al (2) reported prepubertal gynecomastia in 29 out of 581 (5%) patients with gynecomastia (22 bilateral, 7 unilateral). Of these 29 patients, a diagnosis of hyperaromatase syndrome was considered only in 2 patients (6.8%) with bilateral prepubertal gynecomastia. Etiological work-up in the remaining 27 (93.2%) patients, including seven unilateral prepubertal gynecomastia, did not show any underlying pathology and the cases were considered as idiopathic prepubertal gynecomastia (2).

Hyperaromatase syndrome [aromatase excess syndrome (AEXS)], an autosomal dominant disease, characterized by increased aromatase activity and extraglandular aromatization of androgens to estrogen, is a familial form of gynecomastia (9,10). The underlying genetic mechanism is genomic rearrangements at chromosome 15q21 leading to the overexpression of CYP19A1 (aromatase excess). AEXS causes bilateral prepubertal gynecomastia. Our cases had unilateral gynecomastia and did not have a history of familial gynecomastia. Nor did they have any evidence of acceleration in bone age and somatic growth, increased estrogen level or estrogen/androgen ratio, findings which are present in the hyperaromatase syndrome (9,10).

In obese subjects, increased aromatase activity in adipose tissue may lead to aromatization of androgens to estrogens and causes gynecomastia (2). In a study by Einav-Bachar et al (2), 31% of patients with prepubertal gynecomastia were reported to be obese, while in our study, neither of our cases were obese.

Apart from systemic diseases (estrogen-/androgen-secreting tumors, liver dysfunction, hyperthyroidism, etc.) that increase estrogen production or decrease its metabolism, exposure to drugs and chemical products with systemic, autocrine or paracrine estrogenic or antiandrogenic effects can cause prepubertal gynecomastia (1,2,8,11,12). Felner and White (13) reported three prepubertal boys with bilateral gynecomastia due to indirect exposure to estrogen via their mother using a topical estrogen cream. In all three patients, gynecomastia regressed after cessation of use of this cream by the mothers. Henley et al (8) reported bilateral gynecomastia in three prepubertal boys due to application of lavender oil and resolution of the gynecomastia after removal of the causative agent. Our patients had no history of systemic disease or exposure to any chemical products.

It is possible that the overexpression of estrogen receptors in the mammary gland increases the end-organ sensitivity and facilitates the development of gynecomastia in individuals who have low normal circulating estrogen levels (1,2,14). In our cases, serum estrogen and estrogen/testosterone levels were within the normal range, but the noteworthy presence (3+) of estrogen receptors in 100% of periductal epithelial cells suggested increased local estrogen sensitivity. A karyotype analysis from resected breast tissues which could have given us a diagnosis of a localised chromosomal mosaicism causing intensive estrogen receptor expression would reasonably help us to elucidate the etiology of the increased presence of local estrogen receptor. These investigations require a skilled expertise in sampling and there is every chance of underdiagnosis of a mosaicism if the material collected is from the adjacent normal tissue. In addition, we had no opportunity for this investigation due to lack of adequate laboratory facilities. Indeed, Andersen et al (4) showed a 47,XXY mosaicism and presence of 10% estrogen receptor positive cells in the breast epithelial cells of a 3-year-old boy presenting with unilateral gynecomastia. On the other hand, in this patient, karyotype analysis from peripheral blood showed a normal 46,XY chromosomal pattern. The authors suggested that the X-chromosome aneusomy found in the excised breast tissue may have caused an autonomous proliferation in the breast tissue (4). Although we could not perform a karyotype analysis in breast tissue in our cases, immunohistochemical staining showed presence of estrogen receptors in 100% of periductal epithelial cells.

Although rare in the pediatric age group, Sertoli cell tumors of the testes may present with bilateral prepubertal gynecomastia in about 5% of patients (11). Primary tumors of the breast tissue that caused unilateral prepubertal gynecomastia have also been reported (15,16). Hormonal, radiological and histopathological investigations in our two patients did not show any hormonal or tumoral pathology in their testes or breast tissues.

Overall, the main mechanisms that can explain the etiopathogenesis of gynecomastia in male subjects can be summarized as: i) increased estrogen levels due to endogenous excessive estrogen production or systemic exposure to exogenous estrogen, ii) shift in the estrogen/androgen balance in favor of estrogen levels, iii) local paracrine and autocrine estrogenic effect due to local application of estrogen-containing products, iv) increased estrogen sensitivity of the breast tissue and v) primary tumoral lesions of the breast tissue. Of the above, increased estrogen levels and shift in the estrogen/androgen balance predominantly cause bilateral gynecomastia, whereas local exposure to estrogen, primary cystic and solid tumoral masses and increased end-organ sensitivity are more likely to cause unilateral gynecomastia. However, the exact mechanism and etiology of unilateral prepubertal gynecomastia still remain obscure. The underlying etiological mechanism in our two cases is more likely to be the increased local estrogen sensitivity due to increased expression of estrogen receptors in periductal epithelial cells.

Finally, although unilateral prepubertal gynecomastia is generally considered as an idiopathic benign condition, further investigations are needed to clarify the pathogenesis of this condition. All patients with unilateral prepubertal gynecomastia should have a full endocrine and oncologic evaluation. Once a diagnosis of idiopathic unilateral gynecomastia is considered, surgical excision may be the mainstay of therapy.

## Figures and Tables

**Table 1:**
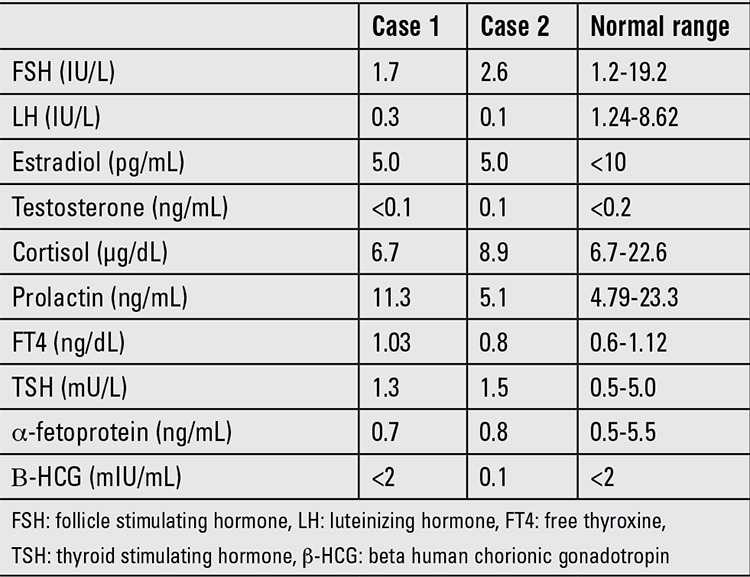
Hormonal evaluation of the two patients
